# Research on risk assessment of cruise tourism supply chain based on catastrophe theory

**DOI:** 10.1371/journal.pone.0306927

**Published:** 2024-08-08

**Authors:** Shengjun Gan, Qingliang Liu

**Affiliations:** School of Economics & Management, Shanghai Maritime University, Shanghai, China; Universidad de Sevilla Facultad de Ciencias Económicas y Empresariales: Universidad de Sevilla Facultad de Ciencias Economicas y Empresariales, SPAIN

## Abstract

The past COVID-19 outbreak caused a huge impact on China’s cruise industry. Now that China’s cruise industry is about to recover, how to assess the risks faced by the cruise industry has become an important issue. On this basis, this paper constructs China’s cruise tourism supply chain and supply chain risk assessment system based on the research contributions made by previous researchers, evaluates the risk indicators of China’s cruise tourism supply chain based on the catastrophe theory, and derives the risk assessment results through the catastrophe progression method, which can be used as a reference for the safe operation of cruise lines in the future.

## 1 Introduction

The modern Chinese cruise industry got its start in 2006, with Costa Cruises opening its first cruise itinerary homeported in China. After a decade of rapid development of China’s cruise industry from 2008–2017, China has become the world’s second largest source country for cruise passengers. The rapid development of the cruise industry at the same time, drives the rapid development of the coastal economy, the formation of various port centers, but this rapid development is also accompanied by many risks, the safety of cruise operation is also increasingly serious problems. Since the outbreak of the COVID-19 epidemic in 2019, which has brought a huge impact on China’s cruise industry, it has become urgent to assess the risks faced by the cruise tourism supply chain, and the cruise tourism supply chain itself is highly vulnerable to risks, so it is worth studying the risk issues for the cruise tourism supply chain.

By reading the literature related to cruise ship risk studies, it is found that most of the literature focuses on a particular type of risk study of cruise ships [1–3], or the risk study of a particular subject in the cruise tourism supply chain [4]. At present, only a few scholars have assessed the risks faced by the current cruise supply chain as a whole from the perspective of the cruise supply chain as a whole [5, 6], and have not yet assessed the risks faced by the supply chain at the level of the individual entities that make up the supply chain. Therefore, this paper takes the cruise tourism service as a starting point and stands in the perspective of the cruise company to assess the risks faced by each subject in the Chinese cruise tourism supply chain.

To this end, this paper asks the following three questions: (1) What is the composition of China’s cruise tourism supply chain? (2) What are the risk indicators faced by each member of China’s cruise tourism supply chain? (3) How are the values of these indicators measured and which risk indicators have higher impact ratings? The rest of the sections are organized as follows: part 2 is the literature review; part 3 is the construction of the risk assessment index system of the cruise tourism supply chain; part 4 introduces the research methodology on which this paper is based; part 5 is the empirical analysis of the cruise tourism supply chain in China; part 6 is the results and discussion; and part 7 summarizes the conclusions of this paper.

## 2 Literature review

### 2.1 Cruise supply chain

Cruise industry is an integrated industry integrating tourism, shipping, leisure and tourism [7], so its supply chain research also involves many aspects, and the existing research mainly focuses on the following two aspects:

First, the main composition of the cruise supply chain. Simon Ve´ronneau et al. (2009) started from a supply chain management perspective, arguing that the supply chain was composed to satisfy customer demand, and summarized that the cruise supply chain started with the cruise line’s suppliers, and ended with the ship itself as the consumer [8]. Xu Hong et al. (2010) argued that the cruise tourism supply chain was centered on the cruise line, forming a chain that includes suppliers of cruise product supply, cruise material supply, etc., to distributors, retailers, and ultimately to tourists [9]. Christos Tsourakis (2012) analyzed the complexity of the cruise supply chain and described the outsourcing of the cruise supply chain through a cruise supply chain consisting of suppliers, port agents, and cruise lines [10]. Heying Sun et al. (2019) argued that the cruise supply chain consisted of multiple aspects involving cruise terminals, cruise lines, and cruise suppliers (e.g., travel agents, airlines, hotels, etc.), with travel agents being a key node connecting tourists, cruise homeports, and cruise lines, and used this as the basis for developing a supply chain system consisting of multiple cruise homeports, a travel agent, and a cruise line [11]. Chenrui Qu et al. (2020) considered the cruise supply chain to be a combination of a product supply chain and a service supply chain, and in this way defined a cruise supply chain system that included the cruise supplier, the cruise line, and the passengers, where the cruise line provided customized cruise travel itineraries for the passengers, and the cruise supplier was the one that provided the necessary supplies for the cruise ship [12]. Jingen Zhou et al. (2022) gave a definition of the cruise supply chain based on the unique characteristics of cruise shipping and by comparing it with the maritime supply chain and the tourism supply chain: it was a network of entities engaged in three phases (distribution services, port services, and on-board and on-shore services) through the provision of cruise products, financial and information services to cruise passengers [13].

Second, a study on the relationship between upstream and downstream in the cruise ship supply chain. Zhang Xiaoli et al. (2015) explored the resource allocation mechanism in the cruise logistics supply chain by constructing a supply chain composed of tourism enterprises, port enterprises and cruise enterprises [14]. Miaoqing Shi et al. (2018) explored the distribution of benefits between cruise lines and cruise agents through a secondary tourism supply chain consisting of one supplier-cruise ship owner and two competing retailers-cruise agents [15]. Zhang Rui et al. (2019) explored the cultivation path of internationalized cruise ship talents from the demand and cultivation status of cruise ship crew members [16], And Yang Xue et al. (2020) analyzed China’s cruise talent supply from the cruise demand supply chain and cruise supply chain aspects, and pointed out certain directions for China’s talent supply [17]. Zhang Yezhen et al. (2020) took the cruise destination as the starting point and construct a cruise supply chain with the cruise homeport as the core based on the upstream and downstream relationships of the cruise industry: from the port city to the cruise homeport to the cruise line to the final tourist [18]. Angela Mai Chi Chu et al. (2021) combined the actual situation in China with the tourism supply chain as a model, and combined with the principal-agent theory, to redefine the cruise tourism supply chain formed by cruise companies, travel agencies and cruise passengers [4]. While Jean-Paul Rodrigue et al. (2022) defined the process of replenishment of cruise ships during their short stay in ports as cruise supply chain management based on the characteristics of cruise shipping, as well as the cruise supply chain relied on its main players, such as cruise ports, cruise lines, and cruise service providers [1].

Summarizing the existing research, cruise supply chain research has basically formed by the cruise company as the core, based on the cruise port, cruise service supply as the auxiliary main framework, the formation of cruise suppliers, cruise companies, cruise passengers mainly cruise supply chain system.

### 2.2 Cruise risk assessment

Although it is safer to travel by means of a cruise ship, the large number of passengers it carries and the complexity of the nationalities of the tourists make it very easy to cause a great disaster in the event of an accident [19]. Therefore, safety assessment of cruise ships has become an issue that cannot be ignored, and the existing research on cruise ship safety mainly includes:

First, research on cruise risk assessment methods. P. Lois et al. (2004), through a critical evaluation of the Formal Safety Assessment (FSA) for cruise ships and a test case study, concluded that there was still room for improvement in cruise ship safety, especially in terms of human factors [20]. Birnur Ozbas (2013), through a review of the literature on safety risks in maritime transportation, argued that compared to the risk of accidents in transportation at sea, the risk was greater when the vessel was in a port, approaching land, or navigating through narrow waterways, and that ports and shipping lanes were the site of major accidents [21]. Sun Xing et al. (2018) started from the three factors affecting the safety of cruise ship navigation: human factors, ship factors, and environmental factors, constructed an index system for evaluating the safety of cruise ship navigation, and took Qingdao Port as an example to combine the cloud model for the evaluation of the safety of cruise ship navigation [22]. Zhang Junhui et al. (2020), in order to avoid the spread of COVID-19 on cruise ships, constructed an assessment model of the risk of COVID-19 infection among large cruise ship personnel by combining the Bayesian network structure to provide a theoretical and practical method for the prevention and control of epidemics [23]. Shuhan Meng et al. (2023) constructed a risk assessment index system for China’s cruise supply chain, combined with set-pair analysis to judge the change trend of China’s cruise supply chain risk, and used the SPA-Markov Chain model to predict the future direction of China’s cruise supply chain system, with the intention of providing countermeasures for the prevention and control of cruise supply chain risk [6].

Second, research on cruise risk. Clare Bowen et al. (2014) identified several risks to cruise ships while at sea by examining the level of terrorism perceived by cruise passengers, with the greatest risk coming from terrorist attacks on ships or ports by extremist groups [3]. Joan P. Mileski et al. (2014) studied 580 cruise ship accidents between 1983 and 2013 and identified four causes of cruise ship accidents: ship design flaws, crew errors, lack of ship maintenance, and unknown causes [24]. Peter Vidmar et al. (2015) used a variety of methods to analyze the risk of cruise ship voyages by examining the different types of accidents that occurred in ports, such as collisions, groundings, and fires, and found that collisions were among the highest risks [25]. Nan Zhang et al. (2016) used a cruise ship as a study to analyze the spread of norovirus on a cruise ship [2]. Yue Jiao et al. (2020), through a critical review of 48 cruise ship accidents that occurred in Asia, concluded that 95.8% of cruise ship accidents were due to human factors, while only 4.2% of the accidents were due to severe weather conditions [26]. Michaela Ibrion et al. (2021) identified the following common safety issues for cruise ships: power outages and sea drifting, fires, collisions, sinkings, groundings, virus transmission, public health and crime [27]. Jingen Zhou et al. (2023), through semi-structured telephone interviews, summarized the risks faced in the cruise supply chain to include: macro risks (natural disasters, climate and weather issues, regulatory risks, macroeconomic risks), safety and health risks (infectious diseases, ship accidents, terrorist attacks, crime and violence, crew overboard, piracy, etc.), information risks (insufficient, inaccurate, and information security, etc.), and supply risks (poor service provided by ship suppliers, ports, inland destinations, and inability to supply in a timely manner, etc.). (insufficient information, inaccurate information, information security, etc.), supply risks (poor quality of service from ship suppliers, ports, inland destinations, and inability to supply on time) [28].

Third, research on the safe operation of cruise ships. Huang Mei (2016) analyzed the advantages and disadvantages of China’s unique form of cruise tourism ‐ the charter mode ‐ and put forward suggestions for improvement based on the perspective of travel agencies [29]. Guo Ping (2016) cut in from a legal perspective, analyzed the problems in China’s cruise ship manufacturing industry, cruise ship service industry, and cruise ship transportation industry, and proposed solutions from a legal perspective [30].

To summarize, scholars’ research on cruise risk only focuses on a single subject or a single risk factor, and very few of them study its risk based on the perspective of the whole cruise supply chain. While the cruise industry is a complex industry cluster involving multiple participating subjects, there is an urgent need for a complete set of research methods to explore and maintain the stable and healthy development of each enterprise in the whole supply chain.

### 2.3 Application of catastrophe theory

Chi-Kuo Mao et al. (2009) proposed an informed approach to understand the nature of the recovery process of Taiwan’s tourism industry based on the cusp catastrophe model of catastrophe theory, while exploring and comparing the recovery patterns of Hong Kong, Japanese, and U.S. tourists coming to Taiwan in the wake of SARS and explaining the differences between the recovery patterns of Hong Kong, Japan, and the United States [31]. Shiliang Su et al. (2011), in order to overcome the shortcomings of land ecological security evaluation methods in terms of subjectivity and complexity, constructed a catastrophe model for land ecological security evaluation with the help of catastrophe theory and evaluated the land ecological security status of Shanghai with this model [32]. Wenjun Wang et al. (2011) applied the multi-indicator evaluation method of catastrophe theory to construct an environmental disaster evaluation model for near-shore waters, and applied this model to assess the possibility of environmental disasters in Dalian Bay [33]. Fengshun Yang et al. (2012) constructed an urban water safety assessment model based on the catastrophe theory and improved the assessment model in two aspects to overcome the shortcomings of the traditional catastrophe evaluation method, and at the same time compared the improved catastrophe evaluation method with the fuzzy synthesis evaluation method and verified its advantageousness [34]. Yangpeng Wang et al. (2017) used the cusp catastrophe model in catastrophe theory to construct a risk framework for the railroad system and described the dynamic change process of the railroad system safety, and qualitatively illustrated the reasonableness of the framework model through five characteristics [35]. Mansour Kheirizadeh Arouq et al. (2020) evaluated the impact of earthquake disaster on urban spatial vulnerability based on the evaluation method of catastrophe theory and combined with the background of geographic information system [36].

Through the literature, it is found that catastrophe theory has been widely used in systematic risk assessment, but it is still not found that scholars use the evaluation method of catastrophe theory to assess the risk of the cruise supply chain system, so this paper intends to adopt the catastrophe theory to study the risk in the cruise tourism supply chain in China.

## 3 Model construction

### 3.1 Cruise tourism supply chain

The concept of value chain was introduced in 1985 by Michael Porter, who argued that "firms are a collection of activities in the process of designing, producing, selling, delivering and supporting their products." All these activities in a business can be represented by a value chain. While there is a value chain behind every cruise activity and it includes the port, the destination, the transport company, the destination management company, the catering provider and so on [37]. Currently, the real competition is no longer between enterprises, but between supply chains and supply chains [38]. While the supply chain as a mesh structure consists mainly of suppliers, producers and distributors, the tourism supply chain is composed mainly of products, distributors and resources [39]. The cruise tourism supply chain, as a type of tourism supply chain, contains these factors in itself.

When combing through the literature on cruise supply chain, the authors found that the current research on cruise supply chain presents diversification. There are supply chains based on cruise ports, in which the research focuses on the outsourcing of cruise supplies, resource allocation and the impact of subsidized participants in the supply chain on cruise homeports [10, 11, 14, 18]; there are supply chains based on cruise companies, in which the research focuses on the composition of the supply chain, the optimization of cruise supply chain, the impact of the disruption of ship supply and other issues [1, 4, 9, 12, 13, 15]. Different scholars build different cruise supply chains based on different problems and from different perspectives. In this paper, we take the cruise company as the main body, cut in from the perspective of tourism, and construct China’s cruise tourism supply chain.

**Fig 1:**
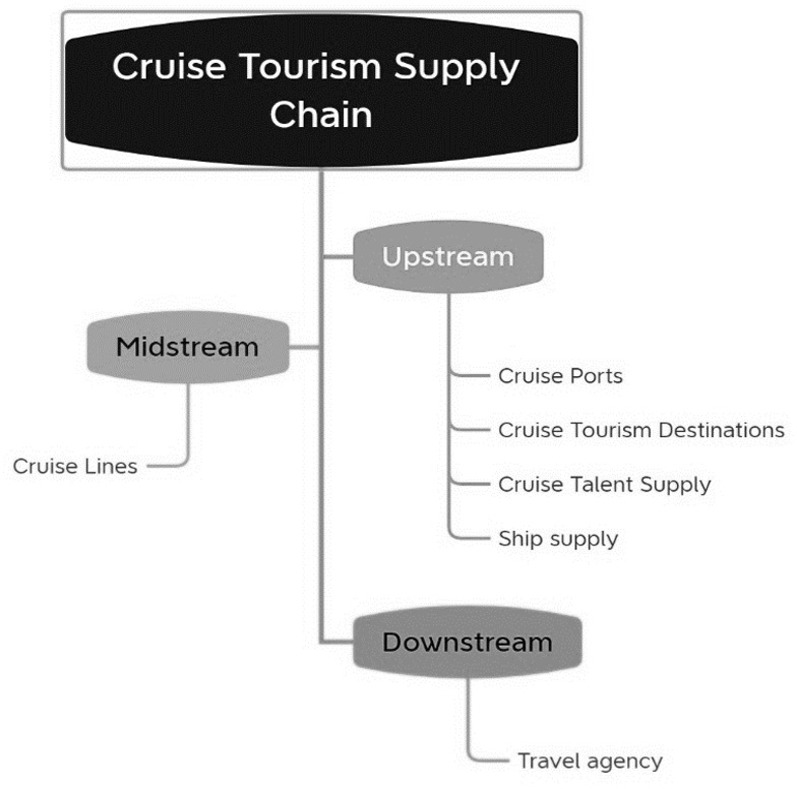
Cruise tourism supply chain.

This paper considers that the cruise tourism supply chain is a kind of cruise supply chain, which is a form of supply chain that is premised on the provision of tourism products/services and aims to meet the wishes of cruise passengers. Based on the definition of supply chain [40], this paper defines that the cruise tourism supply chain refers to the cruise company as the core, relying on cruise ports and cruise destinations to form the cruise tourism products and cruise routes, while the ship supply enterprises and the cruise talent supply system for cruise tourism to provide the necessary cruise supplies and human resources, and finally the travel agency will sell these products/services to cruise passengers. In the cruise tourism supply chain, cruise ports, cruise destinations, ship supply enterprises and cruise talent supply system act as the "suppliers" of the cruise companies, providing the necessary cruise tourism resources for the cruise companies, and the travel agencies act as the "distributors" of the cruise companies, responsible for selling the cruise products and services to the cruise travelers. Cruise ports, cruise destinations and ship supply enterprises, as suppliers to cruise lines, provide cruise lines with berths, on-shore tourism resources and necessary food, beverages and hotel supplies on board, and at the same time, the supply of cruise ship talents provides cruise lines with management and seafaring talents. The travel agency, as a distributor of the cruise line, is the voice of the cruise line to the cruise passengers. The main role of the travel agency is to integrate the food, lodging, tours, shopping, and entertainment provided by the cruise line and its suppliers into the cruise travel supply chain, so as to provide cruise passengers with a good cruise travel experience [41]. Based on this, the system structure of the cruise tourism supply chain is given with the flow of cruise services as the starting and ending points: the upstream includes cruise ports (providing berths for cruise ships and cruise tourism routes), cruise tourism destinations (involving shore excursions for cruise travelers), ship supply enterprises (providing supplies for cruise ships), and the supply of cruise personnel. Midstream for the cruise company. Downstream is the travel agency. Specific as shown in Fig 1.

### 3.2 Cruise tourism supply chain risk assessment indicator system

The cruise industry is highly susceptible to various risks that affect the normal operations of its supply chain members due to its low flexibility and the environmental uncertainties it faces [13, 38],such as environmental uncertainties affecting cruise itineraries and berthing, and the low flexibility of the supply chain affecting ship supply. Cruise tours are mainly divided into ship excursions and shore excursions [42], Cruise ship as a floating sea resort, the ship excursion is the main form of tourism for cruise passengers, the port service supply is to ensure that the ship excursion of the prerequisite and any one of these problems will have an impact on the cruise tourism. At the same time, it is the cruise line’s responsibility to improve the satisfaction of passengers on board and to ensure that the safety and health of personnel on board. In previous studies, personnel operation error is a major factor threatening the safety of cruise tourism [24, 26], which emphasizes the importance of the "human" factor in the cruise tourism supply chain, so this paper includes the supply of cruise personnel in the supply chain risk evaluation index system. Shore excursion is also an important part of cruise tourism, and the safety and security of passengers on shore excursion are closely related to the level of security in the place, and the security risks in the destination also have a certain impact on the safety of passengers [43]. Due to China’s national conditions and unique cruise product sales channels, cruise tourism mainly relies on a few large foreign cruise companies, which rely on the sales model of chartered ships/cabin cuts of travel agencies to transfer the risk, and at the same time, this approach also makes the formation of the low price model of cruises in China [44], which on the one hand, brings a bad experience to cruise travelers, and on the other hand, also makes other cruise companies deterred, and even withdraw from the Chinese market. Based on this, this paper also includes destination policing risk and cruise line competition risk in the assessment index system as well. Finally, through the literature summary and combined with previous research results, the following security assessment index system is given with the cruise tourism supply chain model proposed in this paper, as shown in Table 1.

**Table 1:** Cruise tourism supply chain risk assessment indicators.

Primary indicator	Secondary indicator	Tertiary indicator	Meaning	Source
Upstream risk	Port risk	Cruise ship berthing risk [1]	Ports where cruise ships are unable to dock due to natural factors such as earthquakes, hurricanes, etc.	Jean-Paul Rodrigue et al. (2022)
		Geopolitical risk [28]	Unavailability of cruise ships due to political factors	Jingen Zhou et al. (2023)
	Cruise destination risk	Destination policing risk [43]	Whether the security situation at the destination is conducive to the safety of the passengers	Zou Yongguang et al. (2012)
		Destination service quality risk [4]	Whether the services provided at the destination meet passenger needs	Angela Mai Chi Chu et al. (2021)
	Cruise talent supply risk	Cruise management talent supply risk [17]	Whether the supply of cruise senior management personnel is sufficient	Yang Xue et al. (2020)
		Cruise ship seafarer supply risk [16, 17]	Whether the supply of seafarer is sufficient	Zhang Rui et al. (2019),Yang Xue et al. (2020)
	Ship supply risk	Risk of ship supply disruption [1]	Unforeseen circumstances resulting in interruption of ship supply or poor quality of ship supply	Jean-Paul Rodrigue et al. (2022)
		Ship supply policy risk [30]	Risks due to ship supply policies or tariff barriers	Guo Ping (2016)
Midstream risk	Cruise operation risk	Onboard service quality risk [8]	Whether the services provided on board cruise ships meet the needs of passengers	Simon Ve´ronneau et al. (2009)
		Crew operation risk [20, 26]	Accidents caused by crew errors	P. Lois et al. (2004),Yue Jiao et al. (2020)
		Cruise line competition risk [44]	Risks due to competition among cruise lines	Sun Rui Hong et al. (2012)
	Cruise ship accident risk	Risk of epidemic transmission [2, 28]	Mass infections of persons on board due to the spread of infectious diseases and epidemics	Nan Zhang et al. (2016),Jingen Zhou et al. (2023)
		Risk of terrorist attack [3]	Risk of terrorist attacks on maritime navigation	Clare Bowen et al. (2014)
		Shipboard accident risk [25, 27]	Risk of accidents such as fires, people falling into water, etc.	Peter Vidmar et al. (2015), Michaela Ibrion et al. (2021)
Downstream risk	Travel agency risk	Principal-agent risk [4]	Risks between principals and agents due to special sales channels in China	Angela Mai Chi Chu et al. (2021)
		Cruise sales personnel quality risk [29]	Risks due to unprofessional and inadequately trained sales personnel	Huang Mei (2016)
		Cruise ticket sales risk [44]	Risk of under-selling cruise tickets due to special sales model	Sun Rui Hong et al. (2012)

## 4 Methodology

### 4.1 Catastrophe theory

Catastrophe theory was introduced by French mathematician Rene.Thom in 1972 in his monograph Stable Structures and Morphogenesis, which takes discontinuous phenomena as the object of study, and employs mathematical tools such as topology, singularity theory, and structural stability, to study the jump of a certain system from one state to another. The catastrophe progression method is based on the catastrophe theory, through the combination of the method with fuzzy mathematics, the multi-level decomposition of the evaluation objectives, and generates catastrophe fuzzy subordinate function, using the normalization formula for the integrated quantitative calculation, according to the importance of its analysis and assessment and ultimately arrives at a comprehensive evaluation of the results of the method [45]. Cruise tourism supply chain risk assessment is a complex problem, involving more risk factors, and the existence of mutability of risk factors, while the catastrophe theory since its inception, by the security of the scholars of the wide range of concerns, has been widely used in the field of security [46–48].

### 4.2 Catastrophe progression method

1. Constructing a catastrophe assessment indicator system. According to the purpose of the assessment object and its intrinsic mechanism, it will be decomposed into a multi-level assessment system composed of a number of assessment indicators. As there are generally no more than four control variables in a catastrophe system, the corresponding assessment metrics for each layer should not exceed four.
2. Calculation of weights. By calculating the weight of the index, the importance of the index is given. Common methods for calculating weights include: Analytic Hierarchy Process, entropy method, coefficient of variation method, etc.
3. Determine the type of catastrophe for each indicator stratum. The common types of the catastrophe are: Fold Catastrophe, Cusp Catastrophe, Swallowtail Catastrophe, and Butterfly Catastrophe as shown in Table 2.

**Table 2:** Common types of the catastrophe.

Catastrophe type	Number of control variables	Potential function	catastrophe singularity set equation	Normalization formula
Fold Catastrophe	1(*a*)	*f*(*x*) = *x*^3^+*ax*	*a* = -3*x*^2^	$x_{a}=\sqrt{a}$
Cusp Catastrophe	2(*a*、*b*)	*f*(*x*) = *x*^4^+*ax*^2^+*bx*	*a* = -6*x*^2^,*b* = 8*x*^3^	$x_{a}=\sqrt{a},x_{b}=\sqrt[{3}]{b}$
Swallowtail Catastrophe	3(*a*、*b*、*c*)	$f(x)=\frac{1}{5}x^{5}+\frac{1}{3}ax^{3}+\frac{1}{2}bx^{2}+cx$	*a* = -6*x*^2^,*b* = 8*x*^3^,*c* = -3*x*^4^	$x_{a}=\sqrt{a},x_{b}=\sqrt[{3}]{b},x_{c}=\sqrt[{4}]{c}$
Butterfly Catastrophe	4(*a*、*b*、*c*、*d*)	$f(x)=\frac{1}{6}x^{6}+\frac{1}{4}ax^{4}+\frac{1}{3}bx^{3}+\frac{1}{2}cx^{2}+dx$	*a* = -10*x*^2^,*b* = 20*x*^3^,*c* = -15*x*^4^,*d* = 4*x*^5^	$x_{a}=\sqrt{a},x_{b}=\sqrt[{3}]{b},x_{c}=\sqrt[{4}]{c},x_{d}=\sqrt[{5}]{d}$

In the table, *f*(*x*) denotes the potential function of the state variable *x*, and *a*,*b*,*c*, and *d* are denoted as the control variables affecting the state variable. The catastrophe singularity set equation can be obtained by taking the derivation of *f*(*x*), and then the normalization formula can be derived from the catastrophe singularity set equation.
4. Normalization of indicator results. The data representing the results of the indicator are substituted into the normalization formula to perform a quantitative recursive operation on the system, and finally the value of the system’s total catastrophe affiliation function is derived. This step also takes into account the complementary relationship between the indicators, i.e. there are two principles for taking the values: if the role of the indicators is complementary, the average value is taken; if the role of the indicators is non-complementary, the value is taken according to the principle of "taking the smallest out of the large" [49].
5. Comprehensive evaluation based on the normalized results. A comprehensive analysis and evaluation of the entire system is conducted in conjunction with the purpose and final results of the evaluation.

## 5 Empirical analysis

This study selects a total of 17 risk indicators affecting the upstream, midstream and downstream of the cruise tourism supply chain operation to construct an assessment system, designs a matrix scale based on this indicator system, and invites relevant experts in the cruise industry to make a professional judgment on its importance. Each question item is set up using a 5-level scale, while in order to determine the order of each indicator in the potential function of the catastrophe progression model, this study uses the coefficient of variation method to determine the weights of each indicator. The mean, standard deviation, coefficient of variation and weights of the three levels of indicators are calculated based on the scoring results of 56 experts, and the results are shown in Table 3.

**Table 3:** Means, standard deviations, coefficients of variation and weights of the three levels of indicators.

Tertiary indicator	Mean	Standard deviation	Coefficients of variation	weight
Cruise ship berthing risk	4.3929	0.5284	0.1203	0.04598
Geopolitical risk	4.5893	0.4964	0.1082	0.04134
Destination policing risk	2.4643	0.5709	0.2317	0.08856
Destination service quality risk	2.5357	0.6019	0.2374	0.09073
Cruise management talent supply risk	4.375	0.5244	0.1199	0.04582
Cruise ship seafarer supply risk	2.6786	0.6062	0.2263	0.08651
Risk of ship supply disruption	4.5714	0.4994	0.1092	0.04175
Ship supply policy risk	4.5714	0.5345	0.1169	0.04469
Onboard service quality risk	4.5536	0.5016	0.1102	0.04211
Crew operation risk	4.5893	0.4964	0.1082	0.04134
Cruise lines competition risk	2.6250	0.5897	0.2246	0.08586
Risk of epidemic transmission	4.5000	0.5045	0.1121	0.04285
Risk of terrorist attack	2.7143	0.4941	0.1820	0.06958
Shipboard accident risk	4.6250	0.4885	0.1056	0.04037
Principal-agent risk	4.2857	0.5629	0.1313	0.05020
Cruise sales personnel quality risk	4.4107	0.6260	0.1419	0.05425
Cruise ticket sales risk	2.4821	0.5718	0.2303	0.08805

The weights of the tertiary indicators are added together to obtain the weights of the secondary indicators, and so on to obtain the weights of the primary indicators, as shown in Table 4.

**Table 4:** Risk indicator weights and catastrophe types at all levels of the cruise tourism supply chain.

Primary indicator	Catastrophe type	Weight of secondary indicator	Catastrophe type	Weight of tertiary indicator
Upstream risk	Butterfly Catastrophe	Port risk(0.08732)	Cusp Catastrophe	Cruise ship berthing risk(0.04598)
				Geopolitical risk(0.04134)
		Cruise destination risk(0.17929)	Cusp Catastrophe	Destination policing risk(0.08856)
				Destination service quality risk(0.09073)
		Cruise talent supply risk(0.13232)	Cusp Catastrophe	Cruise management talent supply risk(0.04582)
				Cruise ship seafarer supply risk(0.08651)
		Ship supply risk(0.08644)	Cusp Catastrophe	Risk of ship supply disruption(0.04175)
				Ship supply policy risk(0.04469)
Midstream risk	Cusp Catastrophe	Cruise operation risk(0.16931)	Swallowtail Catastrophe	Onboard service quality risk(0.04211)
				Crew operation risk(0.04134)
				Cruise lines competition risk(0.08586)
		Cruise ship accident risk(0.15281)	Swallowtail Catastrophe	Risk of epidemic transmission(0.04285)
				Risk of terrorist attack(0.06958)
				Shipboard accident risk(0.04037)
Downstream risk	Fold Catastrophe	Travel agency risk(0.19250)	Swallowtail Catastrophe	Principal-agent risk(0.05020)
				Cruise sales personnel quality risk(0.05425)
				Cruise ticket sales risk(0.08805)

1. Standardization of raw data. The original data of the study are obtained from the ratings of industry experts based on the five-level matrix scale, and there is no problem of too large a difference in the data outline or too large a range of data values, but the catastrophe progression method requires that the variables need to be valued between [0,1], so it is necessary to standardize the original data. In this study, the data collects in the matrix scale are the scores of various industry experts who judge the importance of the indicators affecting the risk of the cruise tourism supply chain, so all indicators are standardized as positive indicators.

The normalization formula for the positive indicator is:

$$Y_{ij}=\frac{x-x_{min}}{x_{max}-x_{min}}\left(0\leq Y_{ij}\leq 1\right)$$

2. Normalization process. The upstream and downstream indicators are normalized sequentially based on the normalization formula. Taking the upstream secondary indicator "cruise port risk" as an example, the average of the standardized data of its tertiary indicators "cruise berthing risk" and "geopolitical risk" after scoring 56 experts is taken as follows:0.69643、0.58929, According to the results of the weights calculated by the coefficient of variation method, its weight order is: "cruise berthing risk" > "geopolitical risk", and the three-level indicators meet the principle of complementarity, according to the number of three-level indicators, its catastrophe type is Cusp Catastrophe, which can be obtained from its catastrophe series value is:

$$x_{a}=\sqrt{0.69643}=0.83452$$

$$x_{b}=\sqrt[{3}]{0.58929}=0.83838$$

$$X_{port\;risk}=\frac{x_{a}+x_{b}}{2}=0.83645$$

The remaining indicators, in turn, give the catastrophe progression values for the remaining upstream and midstream tertiary indicators.

The downstream three-level indicators "principal-agent risk", "cruise salesperson quality risk" and "cruise ticket sales risk" follow the principle of non-complementarity, and their catastrophe progression values are:

$$x_{c}=\sqrt[{4}]{0.76191}=0.93428$$

$$x_{b}=\sqrt[{3}]{0.80357}=0.92970$$

$$x_{a}=\sqrt{0.24107}=0.49099$$

$$X_{travel\;agency\;risk}=\min\left\{x_{a},x_{b},x_{c}\right\}=0.49099$$

Calculated sequentially upwards, the final value of the catastrophe progression value for each level of the indicator was obtained as shown in Table 5.

**Table 5:** Assessed values of cruise tourism supply chain risk indicators.

Primary indicator	Rank	Secondary indicator	Rank	Tertiary indicator	rank
Upstream risk(0.88939) Complementary butterfly catastrophe	1	Port risk(0.83645) Complementary cusp catastrophe	2	Cruise ship berthing risk(0.69643)	4
				Geopolitical risk(0.58929)	7
		Cruise destination risk(0.56607) Complementary cusp catastrophe	7	Destination policing risk(0.23214)	15
				Destination service quality risk(0.26786)	13
		Cruise talent supply risk(0.67909) Complementary cusp catastrophe	5	Cruise management talent supply risk(0.68750)	5
				Cruise ship seafarer supply risk(0.22619)	16
		Ship supply risk(0.85812) Complementary cusp catastrophe	1	Risk of ship supply disruption(0.57143)	9
				Ship supply policy risk(0.78571)	2
Midstream risk(0.87996) Complementary cusp catastrophe	2	Cruise operation risk(0.71789) Complementary swallowtail catastrophe	6	Onboard service quality risk(0.55357)	10
				Crew operation risk(0.58929)	7
				Cruise line competition risk(0.20833)	17
		Cruise ship accident risk(0.76015) Complementary swallowtail catastrophe	3	Risk of epidemic transmission(0.50000)	11
				Risk of terrorist attack(0.35714)	12
				Shipboard accident risk(0.62500)	6
Downstream risk(0.70070) Complementary fold catastrophe	3	Travel agency risk(0.49099) Non-complementary swallowtail catastrophe	4	Principal-agent risk(0.76191)	1
				Cruise sales personnel quality risk(0.80357)	14
				Cruise ticket sales risk(0.24107)	3

**Fig 2:**
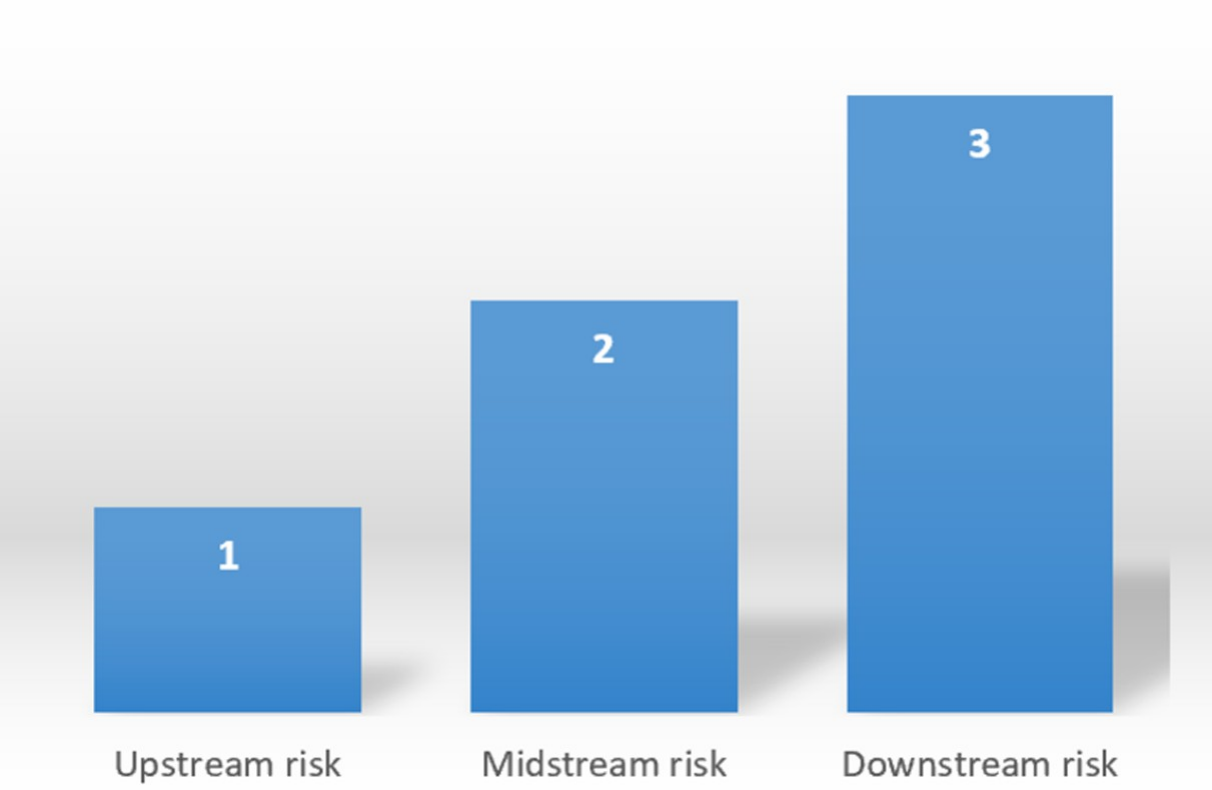
Ranking of assessed values of the primary indicators.

**Fig 3:**
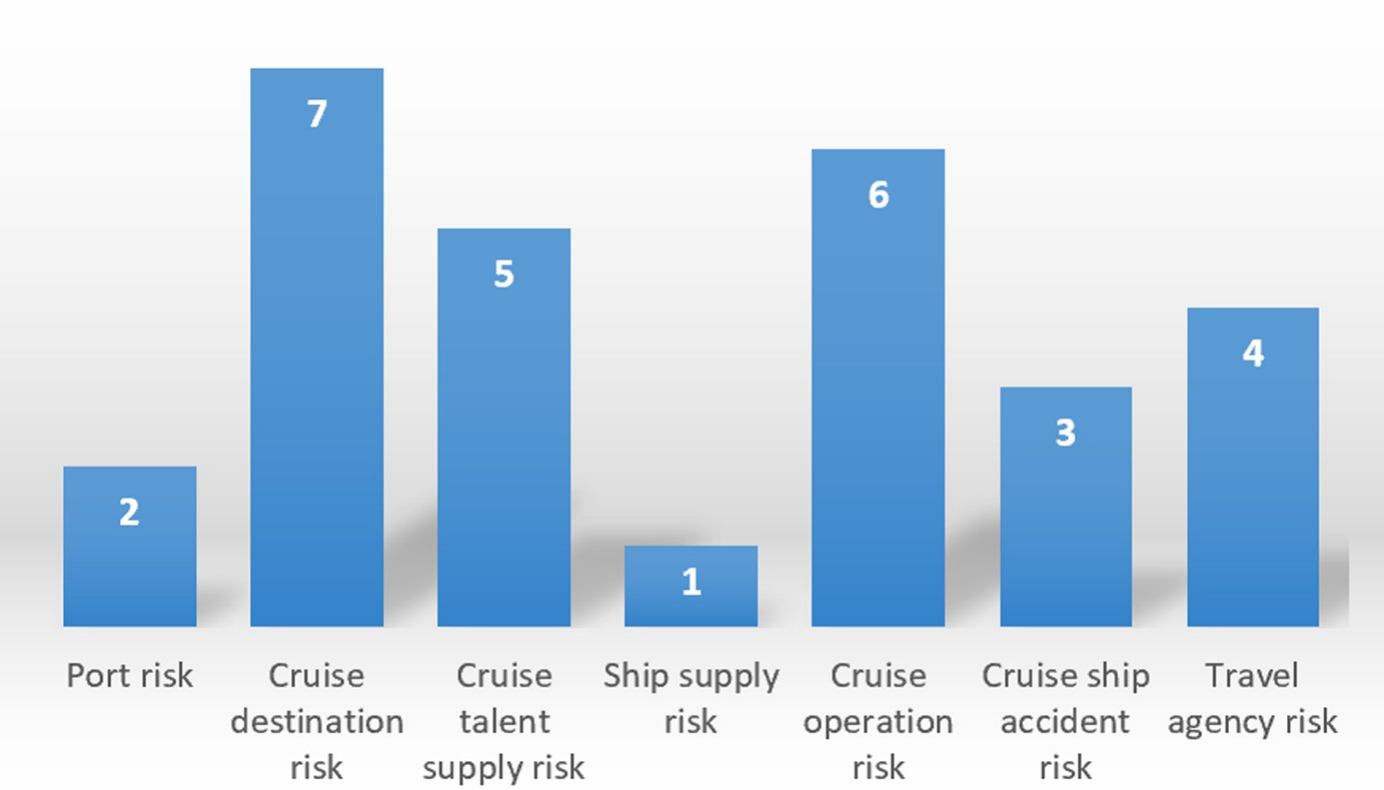
Ranking of assessed values of the secondary indicators.

**Fig 4:**
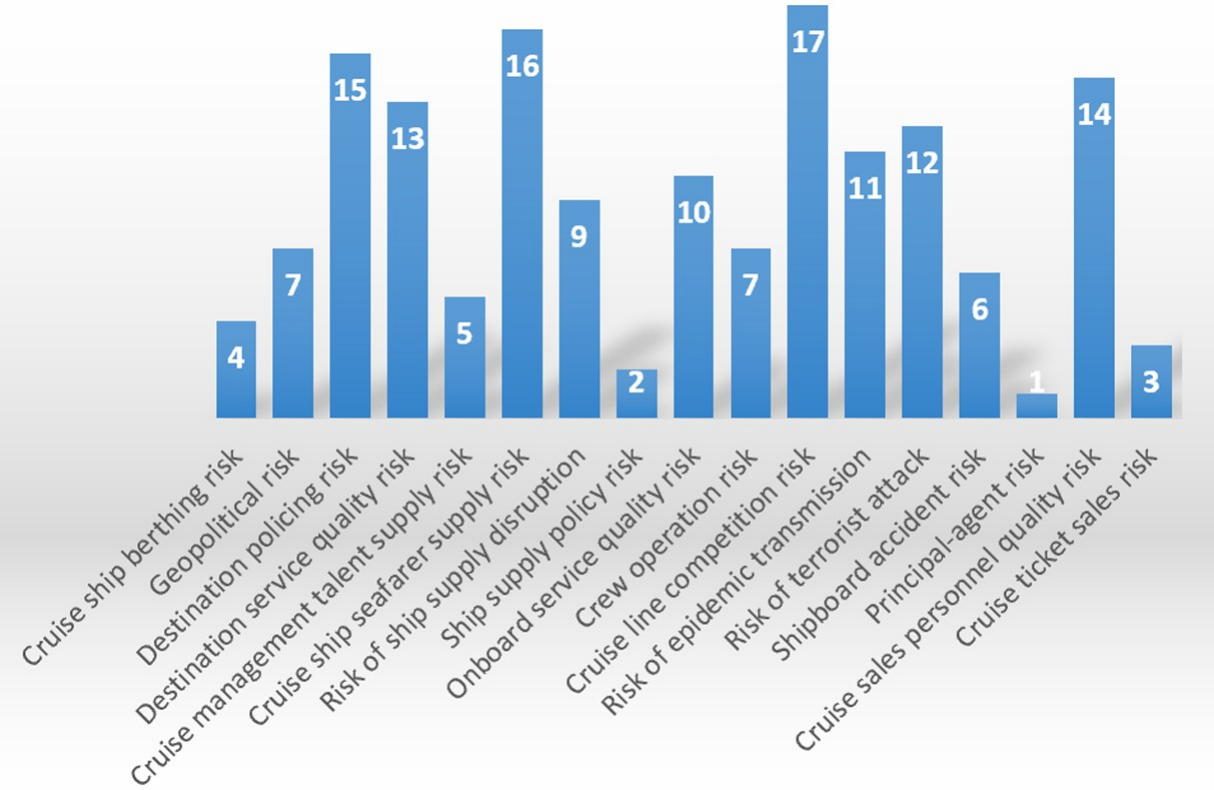
Ranking of assessed values of the tertiary indicators.

The specific results are shown in the following figures: ranking of assessed values of the primary indicators (Fig 2), ranking of assessed values of the secondary indicators (Fig 3), and ranking of assessed values of the tertiary indicators (Fig 4).

## 6 Results and discussion

According to the calculation results in Table 5, the risk indicators for the upstream, midstream and downstream of the cruise tourism supply chain are ranked from largest to smallest: upstream risk, midstream risk, and downstream risk.

Among the upstream risks, "ship supply risk" and "port risk" are the most prominent, and the authors agree with Guo Ping (2016) [30] that China’s cruise industry has experienced more than a decade of rapid development, but the relevant laws and policies have not been synchronized with the update. The cruise industry still faces high taxes and fees, as well as ship supply services cannot enjoy preferential policies such as export tax rebates, resulting in most cruise companies operating in China that do not choose to ship supply in China, which also further enhances the ship supply risk of cruise companies. As for "port risk", on the one hand, as Jean-Paul Rodrigue et al. (2022) [1] argued, most destinations of cruise lines operating in China, especially those homeporting in Shanghai, went to Japan and Korea, which are often plagued by earthquakes, hurricanes, and other disasters, and this causes sometimes cruise ships were unable to stop and call at ports in Korea or Japan; On the other hand, as Jingen Zhou et al. (2023) [28] stated that "the political sensitivity between Northeast Asian countries and regions is high", such as the recent incident of Japan’s discharge of nuclear wastewater, which led to boycotts of Japan by the Chinese public and affected the operation of Japan’s cruise lines. It is worth noting that the "cruise management talent supply risk" is high in the evaluation of the tertiary indicators. China’s cruise industry has been developing for more than ten years, but China’s cruise management talent is very scarce, it is very obvious that the world is almost no Chinese cruise ship captains, how to fill the gap of cruise management personnel is also China’s future development of the cruise industry to face the problem.

Among the midstream risks, "cruise ship accident risk" is higher, which is mainly caused by "shipboard accident risk". As a "sea resort", cruise ships are crowded with people and have strong mobility, which is also easy to cause accidents such as fire and people falling into the water. At the same time, the "risk of epidemic transmission" is also the cause of the high "cruise ship accident risk", which can be seen from the past COVID-19 outbreak, due to the structure of the cruise ship, once an epidemic or infectious disease occurs on board, it will cause widespread infection. In addition, "crew operation risk" and "onboard service quality risk" also contribute to the high "cruise operation risk", one of the reasons is attributed to the high risk of "crew operation error". Previous studies [26] have found that, most of the cruise ship accidents are mainly caused by the improper operation of the ship’s personnel, such as the classic "Costa Concordia" cruise ship reefing accident in 2012, the captain and the first mate were arrested and imprisoned because of this accident. The "charter/cabin" sales model of China’s cruise ships has led to travel agencies "dumping" cabins as a last resort, which is the main reason for the low quality of services on board cruise ships.

Among the downstream risks, "cruise sales personnel quality risk" and "principal-agent risk" are higher, ranking the top three of all three risk indicators. On the one hand, China’s cruise industry has developed rapidly for many years, but the supporting facilities have not kept pace with the development of the cruise industry, and most cruise sales personnel have not fully understood the cruise industry. At the same time, they have not received professional training, which has a negative impact on the popularity of cruise culture and the sales of cruise tickets. On the other hand, as Angela Mai Chi Chu et al. (2021) [4] argued, due to the unique cruise ticket sales model, cruise lines operating in China were only cruise operators, and travel agencies were the real cruise ticket sellers. The travel agency is the "protagonist" who creates cruise market demand for the cruise company, which creates a role bias between the principal (the cruise company) and the trustee (the travel agency).

## 7 Conclusion

This paper constructs a risk assessment system for the cruise tourism supply chain from the perspective of cruise companies, and at the same time develops a new risk assessment method for the cruise supply chain based on the catastrophe theory, which collects data through the expert scoring method and quantitatively calculates the value of the risk of the cruise tourism supply chain based on the catastrophe progression method. The results show that upstream risk is the highest in the overall risk, followed by midstream risk, and downstream risk is the lowest. The results of this study aim to identify the significant risks in the Chinese cruise tourism supply chain and provide certain directions for the safe operation of cruise lines in the future.

In response to the findings of the study, the author believes that improvements can be made in the following areas:

1. Upstream: Relevant departments should accelerate the establishment of ship supply bases in major ports, and at the same time provide tax incentives and legal support for relevant cruise materials, so as to ensure that cruise companies operating in China can smoothly carry out ship supply replenishment in the local area. At the same time, the relevant departments should accelerate the pace of cruise personnel training, and promote colleges and other educational institutions to set up cruise personnel training bases, so as to lay the foundation for China to cultivate excellent cruise personnel.
2. Midstream: Cruise companies should increase the training of the crew, gradually improve the crew’s work quality and proficiency, and improve the quality of service while reducing the occurrence of accidents.
3. Downstream: Relevant departments should gradually improve the relevant laws and relax the restrictions on cruise companies, while cruise companies should also actively expand their ticket sales channels in China, such as setting up joint ventures with qualified local travel agencies, so as to gradually weaken their reliance on commissioned agents.

There are some research shortcomings in this paper, such as due to the limitation of the research method, this paper does not include all the risks faced by the cruise tourism supply chain. At the same time, this paper only evaluates the risks faced by China’s cruise tourism supply chain at this stage, and does not foresee the future development trend of China’s cruise tourism supply chain risks, which is also the author’s next research direction.

## Supporting information

S1 Table Evaluation of the importance of risk indicators in the cruise tourism supply chain. (DOCX)

S2 Table Relevant expert information. (DOCX)

S1 Data (XLSX)
