# Supplementary material for: Research on risk assessment of cruise tourism supply chain based on catastrophe theory
Source: PLoS One. 2024 Aug 8;19(8):e0306927. doi: 10.1371/journal.pone.0306927 (PMC11309406; doi:10.1371/journal.pone.0306927)
Supplement: S1 Table — (DOCX) [file pone.0306927.s001.docx]

S1 Table. Evaluation of the importance of risk indicators in the cruise tourism supply chain: (use of a five-level matrix scale)

| Title/Option | Very unimportant | Unimportant | Medium | Important | Very important |
| --- | --- | --- | --- | --- | --- |
| Cruise berthing risk | 0(0%) | 0(0%) | 1(1.79%) | 32(57.14%) | 23(41.07%) |
| Geopolitical risk | 0(0%) | 0(0%) | 0(0%) | 23(41.07%) | 33(58.93%) |
| Destination policing risk | 0(0%) | 32(57.14%) | 22(39.29%) | 2(3.57%) | 0(0%) |
| Destination service quality risk | 0(0%) | 29(51.79%) | 24(42.86%) | 3(5.36%) | 0(0%) |
| Cruise management talent supply risk | 0(0%) | 0(0%) | 1(1.79%) | 33(58.93%) | 22(39.29%) |
| Cruise ship seafarer supply risk | 0(0%) | 21(37.5%) | 33(58.93%) | 1(1.79%) | 1(1.79%) |
| Risk of ship supply disruption | 0(0%) | 0(0%) | 0(0%) | 24(42.86%) | 32(57.14%) |
| Ship supply policy risk | 0(0%) | 0(0%) | 1(1.79%) | 22(39.29%) | 33(58.93%) |
| Onboard service quality risk | 0(0%) | 0(0%) | 0(0%) | 25(44.64%) | 31(55.36%) |
| Crew operation risk | 0(0%) | 0(0%) | 0(0%) | 23(41.07%) | 33(58.93%) |
| Cruise line competition risk | 0(0%) | 23(41.07%) | 32(57.14%) | 0(0%) | 1(1.79%) |
| Risk of epidemic transmission | 0(0%) | 0(0%) | 0(0%) | 28(50%) | 28(50%) |
| Risk of terrorist attack | 0(0%) | 17(30.36%) | 38(67.86%) | 1(1.79%) | 0(0%) |
| Shipboard accident risk | 0(0%) | 0(0%) | 0(0%) | 21(37.5%) | 35(62.5%) |
| Principal-agent risk | 0(0%) | 1(1.79%) | 0(0%) | 37(66.07%) | 18(32.14%) |
| Cruise sales personnel quality risk | 0(0%) | 1(1.79%) | 1(1.79%) | 28(50%) | 26(46.43%) |
| Cruise ticket sales risk | 0(0%) | 31(55.36%) | 23(41.07%) | 2(3.57%) | 0(0%) |

Note: The numbers in the table represent the number of people who chose that option.
