# Supplementary material for: Research on risk assessment of cruise tourism supply chain based on catastrophe theory
Source: PLoS One. 2024 Aug 8;19(8):e0306927. doi: 10.1371/journal.pone.0306927 (PMC11309406; doi:10.1371/journal.pone.0306927)
Supplement: S2 Table — (DOCX) [file pone.0306927.s002.docx]

S2 Table. Relevant expert information

| Place of work |  | Post/Professional title |  | Work experience |  |
| --- | --- | --- | --- | --- | --- |
| Cruise Line | 10 | Head of Department | 13 | Less than 3 years | 2 |
| Travel Agency | 8 | Executive | 3 | 4-5 years | 6 |
| Ship Supplier | 5 | Senior Staff | 20 | 6-10 years | 30 |
| Cruise Port | 12 | Staff | 8 | More than 10 years | 18 |
| University/College | 9 | Professor/Associate Professor | 5 |  |  |
| Cruise Industry Association | 7 | Others | 7 |  |  |
| Others | 5 |  |  |  |  |
